# The Effect of Tooth Loss on Depression and Anxiety Among Older Adults in China: The Mediating Role of Dietary Diversity

**DOI:** 10.3390/nu18060893

**Published:** 2026-03-12

**Authors:** Yin Wang, Xiaojie Sun

**Affiliations:** 1 Department of Social Medicine and Health Management, School of Public Health, Cheeloo College of Medicine, Shandong University, Jinan 250012, China; wangyin21@mail.sdu.edu.cn; 2 School of Humanities and Management, Ningxia Medical University, Yinchuan 750001, China; 3 NHC Key Laboratory of Health Economics and Policy Research, Shandong University, Jinan 250012, China; 4 Center for Health Management and Policy Research, Shandong University (Shandong Provincial Key New Think Tank), Jinan 250012, China

**Keywords:** tooth loss, depression, anxiety, mediation effect, dietary diversity

## Abstract

**Background/Objectives**: Oral and mental health represent significant public health challenges for the global elderly population. This study aims to explore the association between tooth loss and depression and anxiety symptoms in Chinese elderly individuals, and to assess whether dietary diversity plays a mediating role in this relationship. **Methods**: This cross-sectional study recruited 8413 participants of the 2018 CLHLS. Depression and anxiety symptoms were evaluated with CES-D-10 and GAD-7, respectively. Multivariate logistic regression analysis was used to assess the effect of tooth loss on depression and anxiety symptoms, with adjustment for potential confounding factors. A mediation analysis, based on the PROCESS macro version 4.1, was conducted to further determine whether dietary diversity showed a potential indirect association in this relationship. **Results**: The prevalences of depression and anxiety symptoms were 14.1% and 12.1%. Compared to older adults with 0–8 tooth loss, those with 9–19 tooth loss had higher odds of both depression and anxiety, with odds ratios of 1.388 (95% CI: 1.109–1.614, *p* = 0.002) and 1.248 (95% CI: 1.031–1.512, *p* = 0.023), respectively. Those with 20–27 tooth loss exhibited the highest odds of depression, but no statistically significant increase in anxiety. Complete tooth loss was not significantly associated with either depression or anxiety in the fully adjusted models. Subgroup analysis showed that the association between tooth loss and depression/anxiety symptoms was statistically significant among males, rural residents, those living with family, those with chronic disease, and those without dentures. Mediation analysis suggested that dietary diversity showed a significant indirect association between tooth loss and depression symptoms (β = −0.192, SE = 0.027, 95% CI: −0.245, −0.139, *p* < 0.001), while no significant mediating effect was observed for anxiety symptoms. **Conclusions**: Moderate-to-severe tooth loss correlates with a higher risk of depression and anxiety symptoms in Chinese elderly, with dietary diversity partially mediating the tooth loss and depression association. This finding highlights the need for integrated strategies that combine oral health care, nutritional support, and mental health interventions in the early and middle stages of oral function impairment to protect the mental health of the elderly and improve their quality of life.

## 1. Introduction

Population aging has become a significant challenge worldwide. With an increase in the number and life expectancy of elderly people in China, the mental health problem of the aged is getting worse. Depression and anxiety are the most prevalent mental symptoms in the elderly, serving as critical assessment indicators of their mental health [1,2]. With the growth of the aging population in China, the proportion of depression in the elderly has risen significantly in recent years. Studies suggest that depression symptoms reach 20.0% in Chinese elderly [3], while anxiety disorders are identified as the predominant classification with a lifetime prevalence of 7.6% [4]. They are common and detrimental psychiatric disorders that affect the psychological and social functions of older adults, which are strongly associated with heart and brain diseases [5,6]. Therefore, identifying preventable and modifiable risk factors is crucial for the prevention of these mental illnesses. Meanwhile, the mental health of the elderly is influenced by biological, psychological, and social factors. In particular, health issues closely related to the decline in daily functioning might play a pivotal role in the onset and progression of mental health issues for the aged [7,8]. However, how functional health impairments such as tooth loss modulate depression and anxiety symptoms in the elderly remains underexplored.

Oral diseases have been regarded as a worldwide challenge that affects over 3.5 billion human beings globally, and the cost of treatment is very high, only second to diabetes and cardiovascular diseases [9,10]. Periodontitis, gingivitis, and tooth loss are the common clinical indicators of oral disease or periodontal disease [11]. Among these, tooth loss is widely recognized as the most disabling oral health condition [12], affecting nearly a quarter (23.7%) of the elderly [13]. Tooth loss not only impairs chewing and eating abilities but also potentially exerts a profound impact on the psychological state of the elderly by altering appearance, self-esteem level, and social interaction patterns [14]. From a biopsychosocial perspective, tooth loss is more than a physiological change. It is a multifaceted health condition that involves functional impairment and psychosocial stress across biological, psychological, and social domains. It may influence mental health through multiple interconnected pathways rather than a single mechanism [15]. Complementarily, the oral health–related quality of life (OHRQoL) framework emphasizes that oral conditions are often manifested initially as symptoms and functional limitations, which may be accompanied by downstream psychosocial impacts, including reduced participation and emotional well-being [16]. Consistent with these frameworks, previous studies have reported associations between tooth loss and depression symptoms, with the higher the number of tooth loss, the higher the risk of depression [17,18]. However, most studies treated tooth loss as a linear or dichotomous exposure based on the “functional dentition (≥20 teeth)” [19,20], which overlooks the stages of tooth loss and oral functional decline [21,22]. This approach fails to reflect the impact of varying degrees of tooth loss on the mental health of elderly individuals.

Diet may constitute an important mediating pathway between tooth loss and mental health. Dietary diversity refers to the variety of different foods or food groups that individuals frequently consume. This metric serves as a fundamental measurement for assessing diet quality and is also closely linked to nutritional status [23]. Previous studies indicated that insufficient dietary diversity was not only associated with imbalanced nutrient intake, decreased physical function, and chronic inflammatory states, but also might increase the risk of depression by affecting energy levels, physical activity ability, and emotional regulation mechanisms [24,25]. Within the OHRQoL framework, tooth loss may be reflected in oral symptoms and functional limitations firstly, which constrains food choice and contributes to lower dietary diversity. Meanwhile, the biopsychosocial model provides a broader lens, in which such nutrition-related behavioral changes may co-occur with biological and psychosocial processes relevant to mental health.

Despite growing attention to the links between tooth loss, nutrition, and mental health, several gaps remain. Previous studies primarily focused on the direct association between tooth loss and mental health and rarely examined the dietary pathways that might link oral functional impairment to mental health. Furthermore, tooth loss was often modeled linearly or categorized using functional dentition, which obscured non-linear or stage-specific patterns across different severity levels. Evidence from developing countries, including China, remained limited, despite contextual variations in diet, healthcare access, and social support that could influence these associations.

**Figure 1:**
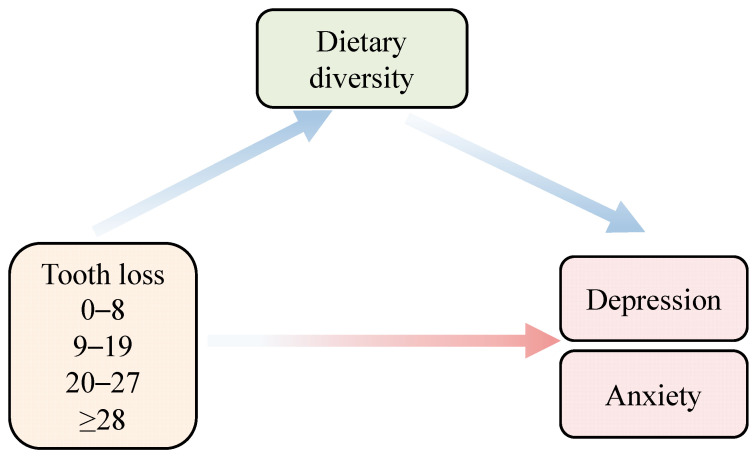
Conceptual model of the association between tooth loss, depression/anxiety and dietary diversity.

To address these gaps, we hypothesized that tooth loss exerted a significant effect on depression and anxiety in Chinese older adults based on the biopsychosocial model and OHRQoL framework. And this impact might display stage-specific characteristics across different tooth loss stages. Furthermore, we posited that dietary diversity mediated the relationship between tooth loss and depression/anxiety symptoms in this population. The conceptual framework is illustrated in Figure 1.

## 2. Materials and Methods

### 2.1. Data Source and Participants

**Figure 2:**
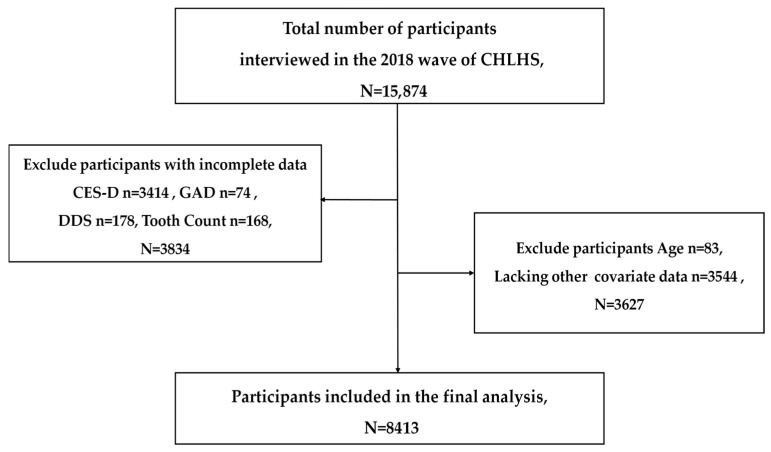
Flow chart depicting the criteria for inclusion and exclusion of the final analytic sample.

The data utilized in this study were collected from a nationally representative survey of Chinese Longitudinal Healthy Longevity Survey (CLHLS), which was established in 1998. The investigation has been performed eight times with track visits every 3 to 4 years. Over 23 provinces were covered by utilizing a multi-stage cluster random sampling method. The CLHLS has been ethically approved by the Biomedical Ethics Committee of Peking University, China (IRB00001052–13074). This cross-sectional study utilized the latest 2017–2018 data, with 15,874 participants. Exclusions were made for participants not completing 10 items on the Center for Epidemiological Studies Depression Scale (CES-D-10), 7 items on the Chinese Generalized Anxiety Disorder Scale (GAD-7), missing data on tooth count and dietary diversity, and individuals under 65 years old with missing or outlier sociodemographic characteristics. Consequently, 8413 participants were included for analysis, as presented in Figure 2.

### 2.2. Assessment of Tooth Loss

The definition of tooth loss refers to the count of missing natural teeth in the mouth, indicating the absence of both natural teeth and any form of restoration [26]. According to diagnostic criteria and recording methods [27], this count is defined as 28 teeth minus the patient’s self-reported number of retained teeth. Based on the functional dentition and previous studies on graded levels of tooth loss [28], participants were categorized into four groups: (1) 0 to 8 tooth loss (indicating minimal loss with largely preserved function); (2) 9 to 19 tooth loss (indicating insufficient functional dentition as a transitional stage); (3) 20 to 27 tooth loss (indicating severe functional impairment); and (4) ≥28 tooth loss (indicating near-complete or complete edentulism). The reliability of self-reported tooth counts has been confirmed by previous studies [29].

### 2.3. Depression

The CES-D-10 was utilized to evaluate the depression in elderly participants through a set of 10 questions focusing on specific emotions or behaviors [30]. The scoring was determined based on the duration of reported negative emotions or behaviors. The CES-D-10 exhibited a Cronbach’s alpha of 0.84, implying satisfactory dependability [31]. Each of the 10 questions was scored on a scale from 0 to 3, with questions 5, 7, and 10 having a reversed scoring pattern. Individuals scoring 10 points or more were classified as having depression [32] based on a criterion supported by various studies on assessing depression in Chinese elderly [33].

### 2.4. Anxiety

The CLHLS utilized the GAD-7 to assess anxiety conditions in the last two weeks [34]. This 7-item analysis employs a 4-point Likert rating system, with responses scored as follows: 0 for never, 1 for occasionally, 2 for more than 7 days, and 3 for every day. With overall scores between 0 and 21, the scale quantifies anxiety severity, where elevated scores correspond to a higher level of anxiety. As observed in previous studies, a score equal to or exceeding 5 signifies the existence of anxiety [35,36].

### 2.5. Dietary Diversity Score

A food frequency questionnaire encompassing 9 types of food, including meat, fish, eggs, beans, sugar, cereals, oil, garlic, and fresh vegetables, was adopted in the CLHLS study to evaluate the Dietary Diversity Score (DDS). Previous studies suggested that dietary diversity score was significantly linked to nutritional status [23]. Since cereals and oils were commonly consumed by Chinese individuals, the consumption of the other seven types of food was used to determine the DDS [22,37]. The consumption frequency of each food category was represented as a binary variable to evaluate the DDS with occasional (0) and daily (1) consumption. As the DDS increases from 0 to 7, the dietary variety becomes more significant [38].

### 2.6. Covariate Variables

Based on previous studies, the anxiety and depression symptoms of older adults might be influenced by demographic characteristics, economic and social characteristics, behavioral and health characteristics, and social support factors [36,39,40]. We adjusted the following confounding variables including demographic characteristics: age (due to significant differences in the trajectory of functional decline among older adults. We divided age into 1 = 65–79, 2 = over 80 years old), gender (1 = male, 2 = female), marriage (1 = married, 2 = others), education (1 = have educated, 2 = uneducated), economic and social characteristics: residence (1 = city or town, 2 = rural), co-residence (1 = with family, 2 = living alone, 3 = live at nursing room), income sources (1 = pensions, 2 = from family members, 3 = others), behavioral and health characteristics: alcohol (yes/no), smoking (yes/no), exercise (yes/no), chronic diseases number (none/1/≥2), denture use (yes/no) and ADL. The CLHLS questionnaire measures an individual’s ADL through six questions, and any item need help is marked as having ADL difficulties (1 = having no difficulty, 2 = having difficulty).

Following the studies based on CLHLS, we operationalized social support through the frequency of social activities and children’s financial support [41,42]. The participants were asked about their recent frequency of joining in social activities, with options including almost every day, not every day but at least once a week, not every week but at least once a month, not every month but sometimes, and not participating. We divided it into 1 = no participation, 2 = sometimes, and 3 = every day. Moreover, we determined whether an individual received financial support from their children based on the question in CLHLS: “How much cash or in kind has been given to you by your children, including all grandchildren and their spouses who live or live separately in the past year?” We divided it into 1 = no financial support from children, and 2 = having financial support from children.

### 2.7. Statistical Analysis

Categorical variables were summarized as frequencies and percentages. Chi-square test was applied to assess disparities in depression or anxiety symptom prevalence for participants. All statistical analyses were performed using SPSS version 27.0, and *p* ≤ 0.05 was considered statistically significant. Logistic regression analyses were conducted to examine the associations between tooth loss and depression and anxiety symptoms, and odds ratios (ORs) with 95% confidence intervals (CIs) were calculated. Three distinct regression models were constructed. Model 1 was a crude model without adjustment. Model 2 was adjusted for demographic and socioeconomic characteristics, including age, gender, marriage, education, residential location, co-residence and income source. Model 3 was further adjusted for health-related and social factors, including smoking status, alcohol consumption, exercise, chronic diseases, denture use, social activity participation, and financial support from children. Subgroup analysis and interaction testing were conducted to examine potential interactions between demographic variables and tooth loss.

The potential mediating effect of dietary diversity on the relationship between tooth loss and depression/anxiety symptoms was investigated using the Hayes macro PROCESS in SPSS [43]. The mediation model controlled for the same set of covariates included in the fully adjusted logistic regression model (Model 3). Bootstrap resampling with 5000 samples was used to estimate the indirect effects. A two-sided *p* value of 0.05 was considered statistically significant, and a mediation effect was considered significant when the 95% confidence interval (CI) excluded zero. Given the cross-sectional design of this study, the mediation analysis was interpreted as reflecting statistical associations rather than causal relationships.

## 3. Results

### 3.1. General Characteristics

8413 participants aged over 65, with 45.64% males and 54.36% females, were evaluated. The prevalence of depression symptoms in the survey sample was 14.10%, and the percentage of anxiety symptoms was 12.09%. In addition, 2415 (28.7%) had 0–8 tooth loss. 1507 (17.9%) had 9–19 tooth loss. 2079 (24.7%) had 20–27 tooth loss, and 2412 (28.7%) had more than 28 tooth loss. A total of 1186 exhibited depression symptoms, of which 909 (76.6%) had 9 or more tooth loss. Similarly, 1017 individuals possessed anxiety symptoms, with 748 (69.2%) having 9 or more missing teeth. Tooth loss, age, gender, marital status, education, residence, co-residence, source of income, smoking, alcohol, exercise, chronic diseases, ADL, denture use, social activities and financial support from children were significant determinants of depression and anxiety symptoms (*p* < 0.05). Detailed information is presented in Table 1.

### 3.2. Association Between Tooth Loss and the Symptoms of Depression and Anxiety

As shown in Table 2 and Table 3, Models 1 and 4 indicated that individuals with 9–19 tooth loss exhibited more depression (OR = 1.338, 95%CI: 1.109–1.614, *p* = 0.002) and anxiety symptoms (OR = 1.248, 95%CI: 1.031–1.512, *p* = 0.023) compared with those having 0–8 tooth loss. Notably, the effect of tooth loss on anxiety was observed significantly in the cases of 9–19 tooth loss. However, individuals with 20–27 and 28 or more tooth loss also showed depression symptoms (OR = 1.495, 95%CI: 1.262–1.772, *p* < 0.001; OR = 1.268, 95% CI: 1.072–1.501, *p* = 0.006). Upon adjusting for socio-demographic factors in Models 2 and 5, the elderly with 9–19 tooth loss continued to be significantly correlated with depression (OR = 1.250, 95%CI: 1.031–1.514, *p* = 0.023) and anxiety symptoms (OR = 1.238, 95%CI: 1.017–1.507, *p* = 0.034). In the fully adjusted models (Models 3 and 6), losing 9–19 teeth remained a significant factor of depression (OR = 1.259, 95%CI: 1.035–1.532, *p* = 0.021) and anxiety symptoms (OR = 1.262, 95%CI: 1.034–1.541, *p* = 0.022).

### 3.3. Relationship Between Tooth Loss and Depression/Anxiety Symptoms in Different Subgroups

In various subgroups, the absence of 9 to 19 teeth significantly correlated with symptoms of anxiety and depression. Among the elderly individuals with 9 to 19 tooth loss, statistical significance was observed in males (OR = 1.470, 95%CI: 1.090–1.983, *p* = 0.012 and OR = 1.498, 95%CI: 1.097–2.045, *p* = 0.011), those having educational experience (OR = 1.398, 95%CI: 1.082–1.807, *p* = 0.010 and OR = 1.339, 95%CI: 1.031–1.740, *p* = 0.029), rural residents (OR = 1.360, 95%CI: 1.083–1.707, *p* = 0.008 and OR = 1.274, 95%CI: 1.017–1.597, *p* = 0.035), those living with family (OR = 1.432, 95%CI: 1.141–1.797, *p* = 0.002 and OR = 1.310, 95%CI: 1.040–1.649, *p* = 0.022), those with chronic diseases (OR = 1.245, 95%CI: 1.007–1.540, *p* = 0.043 and OR = 1.296, 95%CI: 1.042–1.611, *p* = 0.020), and those without dentures (OR = 1.382, 95%CI: 1.102–1.733, *p* = 0.005 and OR = 1.308, 95%CI: 1.306–1.653, *p* = 0.024). Gender (*p* value for interaction = 0.034) and education (*p* value for interaction = 0.011) modified the association between tooth loss and depression. Other subgroup interaction tests revealed that the interaction *p*-value was > 0.05, and there was no statistically significant interaction between subgroup variables and the association of tooth loss with depression/anxiety, as presented in Tables S1 and S2 in the Supplementary Materials.

### 3.4. Mediation Analysis

**Figure 3:**
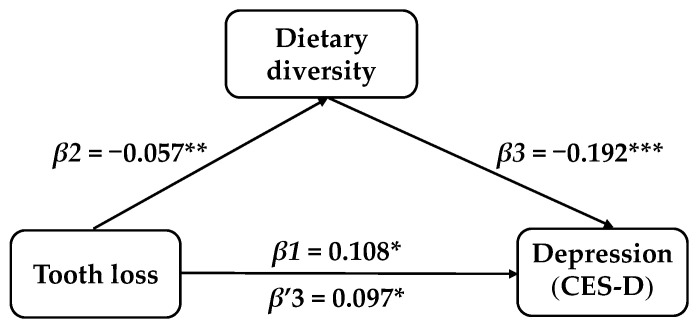
The mediator role of dietary diversity on tooth loss and depression. * *p* ≤ 0.05, ** *p* ≤ 0.01, *** *p* ≤ 0.001.

**Figure 4:**
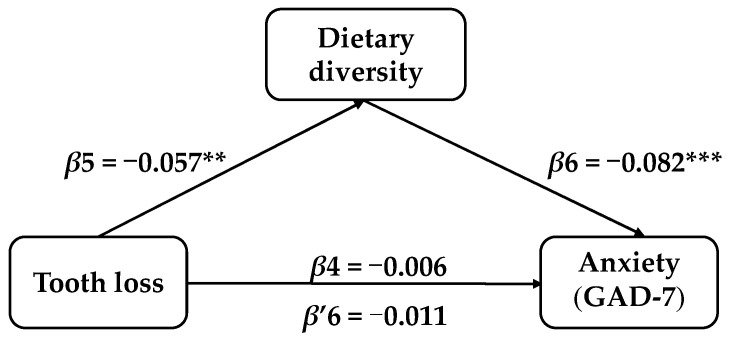
The mediator role of dietary diversity on tooth loss and anxiety symptoms. ** *p* ≤ 0.01, *** *p* ≤ 0.001.

After controlling for the covariates, the mediator role of dietary diversity in the correlation between tooth loss and depression symptoms in Chinese elderly was confirmed. Model 1 indicated that tooth loss exhibited a total effect on depression symptoms in the elderly (*β* = 0.108, t = 2.389, SE = 0.045, *p* = 0.017, 95% CI: 0.019–0.197). The results of Model 2 showed that tooth loss also influenced the dietary diversity scores (*β* = −0.057, t = −3.132, SE = 0.018, *p* = 0.006, 95% CI: −0.093–−0.021). Model 3 implied that after adding the mediating variable, the dietary diversity score had a significant impact on depression symptoms (*β* = −0.192, t = −7.132, SE = 0.027, *p* ≤ 0.001, 95%CI: −0.245–−0.139), while the impact of tooth loss on depression remained remarkable (*β* = 0.097, t = 2.152, SE = 0.045, *p* = 0.031, 95%CI: 0.009–0.186). Tooth loss not only had a direct impact on depression symptoms in elderly people, but also possessed an indirect impact through dietary diversity scores (indirect effect = 0.011, SE = 0.004, 95%CI: 0.004–0.019). The percentage of the total effect mediated by dietary diversity was 10.19%, while the direct influence of tooth loss on anxiety symptoms in elderly people was negligible (*β* = −0.006, t = −0.199, SE = 0.031, *p* = 0.842, 95%CI: −0.066–0.054), as illustrated in Table 4 and Figure 3 and Figure 4.

## 4. Discussion

As far as we know, there was no previous study to explore the correlation between the number of tooth loss and depression/anxiety in the elderly in China. The results imply that tooth loss has a significant impact on the mental health of the elderly in China. For older adults with 9–19 tooth loss, the correlation between tooth loss and the occurrence of depression and anxiety is more pronounced. And these associations are stronger in males, rural residents, those living with family, those with chronic diseases, and those without dentures. Meanwhile, the dietary diversity score plays a critical role in the association between tooth loss and depression in Chinese elderly.

According to the results, the prevalence of depression reached 14.10%. For anxiety symptoms, it was 12.09%, mirroring the rates found among the elderly in China [44]. Among 8413 participants, 28.7% (*N* = 2415) had 0–8 tooth loss, 17.9% (*N* = 1507) had 9–19 tooth loss, 24.7% (*N* = 2079) had 20–27 tooth loss, and 28.7% (*N* = 2412) had 28 tooth loss. Our cohort study data indicated that 71.3% of Chinese older adults had less than 20 teeth, signifying a substantial shortfall to meet the WHO’s criteria for functional dentition. Notably, more than two-thirds of the participants did not have enough teeth to support normal oral function, underscoring a significant public health concern regarding age-related tooth loss in China.

Logistic regression analyses indicated that tooth loss was significantly associated with depression and anxiety symptoms among older adults. Notably, the association with depression exhibited a stage-like pattern, with a marked increase at moderate levels of tooth loss, followed by an apparent plateau at more severe levels. This finding is consistent with the views of the biopsychosocial models and the OHRQoL framework, which posit that oral conditions influence mental health through interconnected functional and psychosocial pathways. Tooth loss can compromise chewing and eating ability, thereby constraining dietary choices. It also influences subjective oral health perceptions, self-esteem, and social interaction pattern factors that are closely linked to emotional well-being [14]. In our study, compared with participants with 0–8 tooth loss, those with 9–19 tooth loss had higher odds of both depression and anxiety. While those with 20–27 tooth loss exhibited the highest odds of depression, but no statistically significant increase in anxiety. Importantly, complete tooth loss was not significantly associated with either depression or anxiety in the fully adjusted models.

This finding differs from prior studies [18,19], and several non-mutually exclusive explanations might account for the observed non-linear pattern. First, the concept of functional dentition provides a clinically meaningful threshold. Having at least 20 natural teeth is commonly used as a benchmark for maintaining basic masticatory function and acceptable appearance [20]. From this perspective, the transition from mild tooth loss (0–8 tooth loss, typically still ≥20 remaining teeth) to moderate tooth loss (9–19 tooth loss, often <20 remaining teeth) may represent a shift from a compensable oral condition to a functionally limiting stage [45]. At this stage, eating restrictions, reduced dietary variety, and increased social-psychological burden may become more salient, thereby elevating vulnerability to depression and anxiety symptoms [46]. Second, at more severe levels of 20–27 tooth loss, individuals may adopt compensatory strategies—such as denture use [47], long-term dietary adaptation, and enhanced caregiving support. These factors may partially alleviate functional limitations and contribute to the apparent plateau in associations observed in cross-sectional analyses. Third, the lack of a significant association with depression or anxiety for complete edentulism in the fully adjusted model may reflect heterogeneity in prosthetic reconstruction and social support, as well as long-term psychological/functional adaptation and survivor/selection bias [48]. Edentulous older adults vary in access to dentures, prosthesis fit, and adherence to denture use. In some cases, effective rehabilitation may partially restore masticatory function and enhance appearance-related confidence and social participation, potentially mitigating psychological distress [47,48]. Moreover, because edentulism is often long-standing, individuals may gradually adapt to functional and aesthetic changes over time. Such long-term adaptation, together with survivor or selection processes in older populations, may attenuate the associations detectable in cross-sectional analyses.

From an adaptive health perspective, this stage-dependent pattern may also be interpreted within the Roy Adaptation Model, which conceptualizes health responses as the product of coping processes under varying stimulus intensities [49]. Tooth loss may function as a chronic stimulus. When it exceeds an individual’s coping ability in certain functional stages, such as beyond the number of functional dental arches, psychological distress may be more likely to emerge [50]. As individuals progress to more severe tooth loss, a new balance may be established through behavioral and environmental adaptations, which may reduce the observable incremental risk [51]. Overall, our findings suggest that the mental health correlates to tooth loss may be better understood as stage-specific effects linked to functional thresholds and adaptation processes rather than as a simple linear accumulation.

Subgroup analyses indicated that the association between tooth loss and depression and anxiety symptoms was more pronounced among males, rural residents, individuals living with family, those with chronic diseases, and those without dentures. Among males, this association might be related to poorer oral health behaviors, including less frequent dental visits and lower engagement in preventive care, which increased the risk of oral diseases and tooth loss [52,53,54]. The association was also stronger among rural residents, which might reflect disparities in their access to dental services and preventive care between urban and rural areas in China [55,56,57]. Limited oral health resources and lower utilization of dental services in rural settings might also contribute to a higher burden of untreated oral conditions among older adults [58,59]. In addition, older adults living with family showed stronger associations between tooth loss and psychological symptoms [60]. Although family support can provide care for older adults, insufficient awareness of oral health or delayed dental intervention may contribute to progressive tooth loss and subsequent psychological stress related to functional limitations or appearance [61,62]. The association was also pronounced among individuals with chronic diseases. This finding is consistent with previous studies suggesting that people with chronic conditions are more vulnerable to psychological distress and may experience poorer oral health outcomes [63]. Finally, the relationship between tooth loss and psychological symptoms was more apparent among individuals without dentures. The absence of dentures may impair chewing, speech, and social confidence, thereby contributing to social withdrawal and psychological distress [64,65,66].

The mediation analysis showed that tooth loss exhibited a negative correlation with dietary diversity scores. Specifically, an increase in tooth loss corresponded to a deterioration in dietary diversity for the elderly. Consequently, a significant indirect association of dietary diversity was observed in depression symptoms, whereas no significant indirect association was observed in anxiety. This difference may be ascribed to the different psychological and neurobiological mechanisms behind the two psychological problems. Anxiety is more closely related to psychological and physiological reactions such as direct threat perception, appearance inferiority, and social pressure [67]. On the contrary, depression is closely related to the decrease in dietary diversity caused by tooth loss [68]. Consistent with previous studies [69], dietary diversity played an important role in the protective mechanism between tooth loss and depression. Our results showed that for every one-point decrease in dietary diversity score, the value of depression symptoms increased by 0.192 points. The intake of a limited range of food means that a person will consume fewer micronutrients and antioxidants, as well as less dietary fiber, which may increase the risk of depression through mechanisms of inflammation, disruption of the microbiota–gut–brain axis, and damage to neurotransmitter synthesis [70].

Different from previous studies [17,18,19], our cross-sectional findings suggested that tooth loss might be non-linearly associated with depression and anxiety symptoms. The elderly with the highest psychological vulnerability experienced moderate tooth loss (9–19). From a functional perspective, this stage may coincide with crossing a functionally meaningful threshold. In this situation, masticatory limitations and related psychosocial burdens become more salient. In addition, our mediation analysis provides mechanistic clues that dietary diversity may partially mediate the association between tooth loss and depression symptoms. Nevertheless, a comparable indirect pathway was not supported for anxiety, implying potentially different pathways for anxiety-related outcomes. This study integrates a threshold perspective with a behavioral–nutritional pathway in a large sample of Chinese older adults. It expands the evidence base from a developing-country context and underscores the value of considering oral function, nutrition, and mental health within an integrated public health framework.

The advantages of this study are summarized as follows. First, with a relatively large sample of community-dwelling Chinese older adults, we demonstrated a potential non-linear relationship between tooth loss and psychological symptoms, emphasizing overlooked stage-specific patterns in previous analyses. Second, the utilization of mediation analysis offered initial insights into the role of dietary diversity in connecting tooth loss to depression symptoms, indicating that depression and anxiety may involve distinct pathways. Moreover, comprehensive adjustment for a range of sociodemographic and health-related covariates enhanced the internal validity of the findings. By presenting population-based evidence from a developing-country context with limited data, this study contributed to a more comprehensive understanding of the correlation between oral health, nutrition, and mental well-being in later life.

Despite the above contributions, this study still has limitations. First, the cross-sectional design precludes causal inference and does not allow us to establish the temporal ordering among tooth loss, dietary diversity, and depressive/anxiety symptoms. Therefore, the reverse causality cannot be ruled out. For example, depression or anxiety may reduce motivations for oral self-care and dental service utilization, and may be associated with adverse health behaviors, which could accelerate oral disease progression and subsequent tooth loss. Although mediation analysis was conducted to explore the potential indirect association through dietary diversity, the cross-sectional nature of the data means that these findings should be interpreted cautiously and cannot be considered evidence of a causal mediation pathway. Second, in this study, tooth loss was measured using self-reported data, not distinguishing between natural teeth and restorations, nor evaluating the number of functional teeth, denture fit, or functional bite units. Including fixed restorations in natural teeth may lead to systematic bias in functional tooth counts [29]. Therefore, the number of teeth in this study should not be equal to the oral functional status. In addition, denture use was measured only as a binary variable (yes/no), and detailed information such as denture fit, functionality, and wearing duration was not available. These factors may influence oral function and comfort and consequently exert differential effects on psychological outcomes. Third, dietary diversity was based solely on the existence of simple indicators of food type and could not reflect nutritional level or actual intake. Also, self-reported indicators by participants may result in recall bias. Finally, the potential mediating roles of factors such as psychological resilience, access to dental care, and dietary nutrient density in the association between tooth loss and mental health were not examined.

## 5. Conclusions

In summary, tooth loss was associated with depression and anxiety symptoms among Chinese older adults. The association, particularly for depression symptoms, exhibited a stage-like pattern, with the highest psychological vulnerability observed at the moderate stage of tooth loss (9–19 tooth loss). While the complete tooth loss was not significantly associated with either outcome in fully adjusted models. Interpreted within the biopsychosocial and OHRQoL frameworks, these findings support the perspective that tooth loss may influence mental health through combined functional limitations and psychosocial consequences. The magnitude of association may differ across stages of oral functional change. In addition, dietary diversity showed a significant indirect effect linking tooth loss with depressive symptoms, providing mechanistic clues for a tooth loss–diet–mental health pathway. However, a comparable indirect pathway was not observed for anxiety. Overall, these findings highlight the potential value of integrating oral function maintenance/rehabilitation, nutrition support, and mental health screening in ageing-related public health practice. Nevertheless, longitudinal and intervention studies are needed to establish temporal ordering and to clarify stage-specific pathways. Future research should prioritize the implementation and rigorous evaluation of dietary diversity interventions for older adults to alleviate the adverse psychological health effects associated with tooth loss.

## Figures and Tables

**Table 1:** General features of depression and anxiety symptoms (*N* (%)).

Characteristic	Depression		*p*	Anxiety		*p*
	Yes	No		Yes	No	
Tooth loos			<0.001			0.006
0–8	277 (11.5)	2138 (88.5)		276 (11.4)	2139 (88.6)	
9–19	227 (15.1)	1280 (84.9)		209 (13.9)	1298 (86.1)	
20–27	338 (16.3)	1741 (83.7)		274 (13.2)	1805 (86.8)	
≥28	344 (14.3)	2068 (85.7)		258 (10.7)	2154 (89.3)	
Age (years)			0.005			0.008
65–79	443 (12.8)	3013 (87.2)		457 (13.2)	2999 (86.8)	
≥80	743 (15.0)	4214 (85.0)		560 (11.3)	4397 (88.7)	
Gender			<0.001			<0.001
Male	452 (11.8)	3388 (88.2)		354 (9.2)	3486 (90.8)	
Female	734 (16.1)	3839 (83.9)		663 (14.5)	3910 (85.5)	
Marriage			<0.001			0.025
Married	466 (11.7)	3520 (88.3)		448 (11.2)	3538 (88.8)	
Others	720 (16.3)	3707 (83.7)		569 (12.9)	3858 (87.1)	
Education			<0.001			<0.001
No	622 (16.9)	3050 (83.1)		544 (14.8)	3128 (85.2)	
Yes	564 (11.9)	4177 (88.1)		473 (10.0)	4268 (90.0)	
Residence			0.003			<0.001
City	264 (12.3)	1900 (87.8)		187 (8.6)	1977 (91.4)	
Rural	933 (14.8)	5327 (85.2)		830 (13.3)	5419 (86.7)	
Co-residence			<0.001			<0.001
With family	868 (12.9)	5884 (87.1)		769 (11.4)	5983 (88.6)	
Alone	259 (18.9)	1111 (81.1)		205 (15.0)	1165 (85.0)	
Nursing home	59 (20.3)	232 (79.7)		43 (14.8)	248 (85.2)	
Income source			<0.001			<0.001
Pensions	278 (10.9)	2262 (89.1)		225 (8.9)	2315 (91.1)	
Family	600 (15.9)	3168 (84.1)		498 (13.2)	3270 (86.8)	
Others	308 (14.6)	1797 (85.4)		294 (14.0)	1811 (86.0)	
Alcohol			<0.001			<0.001
No	1067 (14.9)	6088 (85.1)		906 (12.7)	6249 (87.3)	
Yes	119 (9.5)	1139 (90.5)		111 (8.8)	1147 (91.2)	
Smoking			0.003			0.001
No	1032 (14.6)	6046 (85.4)		890 (12.6)	6188 (87.4)	
Yes	154 (11.5)	1181 (88.5)		127 (9.5)	1208 (90.5)	
Exercise			<0.001			<0.001
No	925 (16.9)	4542 (83.1)		720 (13.2)	4747 (86.8)	
Yes	261 (8.9)	2685 (91.1)		297 (10.1)	2649 (89.9)	
Chronic disease status			<0.001			<0.001
None	174 (9.1)	1745 (90.9)		191 (10.0)	1728 (90.0)	
1	318 (11.2)	2527 (88.8)		358 (12.6)	2487 (87.4)	
≥2	525 (14.4)	3124 (85.6)		637 (17.5)	3012 (82.5)	
ADL			<0.001			0.020
No difficulty	858 (12.6)	5933 (87.4)		793 (11.7)	5998 (88.3)	
Difficulty	328 (20.2)	1294 (79.8)		224 (13.8)	1398 (86.2)	
Denture use			<0.001			<0.001
No	792 (15.7)	4247 (84.3)		676 (13.4)	4363 (86.6)	
Yes	394 (11.7)	2980 (88.3)		341 (10.1)	3033 (89.9)	
Social activities			0.001			0.016
Never	1040 (14.7)	6040 (85.3)		878 (12.4)	6202 (87.6)	
Sometimes	120 (11.1)	959 (88.9)		103 (9.5)	976 (90.5)	
Everyday	26 (10.2)	228 (89.8)		36 (14.2)	218 (85.8)	
Financial support from children			<0.001			0.027
No	645 (12.9)	4373 (87.1)		574 (11.4)	4444 (88.6)	
Yes	541 (15.9)	2854 (84.1)		443 (13.0)	2952 (87.0)	

**Table 2:** Multivariate logistic regression model to analyze tooth loss and depression symptoms.

Characteristic	Reference	Model 1	*p*	Model 2	*p*	Model 3	*p*
		OR [95%CI]		OR [95%CI]		OR [95%CI]	
Tooth loos							
9–19	0–8	1.338 [1.109–1.614]	0.002	1.250 [1.031–1.514]	0.023	1.259 [1.035–1.532]	0.021
20–27	0–8	1.495 [1.262–1.772]	<0.001	1.309 [1.090–1.572]	0.004	1.337 [1.106–1.617]	0.003
≥28	0–8	1.268 [1.072–1.501]	0.006	1.094 [0.909–1.318]	0.341	1.175 [0.957–1.443]	0.123
Age (years)							
≥80	65–79			0.942 [0.804–1.104]	0.460	0.796 [0.674–0.939]	0.007
Gender							
Female	Male			1.188 [1.031–1.368]	0.017	1.099 [0.944–1.279]	0.224
Marriage							
Others	Married			1.125 [0.958–1.320]	0.150	1.032 [0.875–1.217]	0.706
Education							
Yes	No			0.815 [0.702–0.947]	0.007	0.885 [0.760–1.031]	0.117
Residence							
Rural	City			0.964 [0.797–1.165]	0.702	1.002 [0.824–1.219]	0.983
Co-residence							
Alone	With family			1.432 [1.213–1.691]	<0.001	1.649 [1.389–1.958]	<0.001
Nursing home	With family			1.749 [1.289–2.373]	<0.001	1.563 [1.145–2.134]	0.005
Income source							
Family	Pensions			1.368 [1.123–1.667]	0.002	1.274 [1.041–1.559]	0.019
Others	Pensions			1.306 [1.057–1.613]	0.013	1.246 [1.004–1.546]	0.045
Alcohol							
Yes	No					0.713 [0.576–0.882]	0.002
Smoking							
Yes	No					0.989 [0.810–1.207]	0.910
Exercise							
Yes	No					0.550 [0.472–0.641]	<0.001
Chronic disease status							
Yes	None					1.820 [1.538–2.153]	<0.001
ADL							
Difficulty	No difficulty					0.635 [0.541–0.745]	<0.001
Denture use							
Yes	No					0.714 [0.618–0.824]	<0.001
Social activities							
Sometimes	Never					1.019 [0.820–1.267]	0.866
Everyday						1.078 [0.703–1.651]	0.731
Financial support from children							
Yes	No					0.958 [0.706–1.299]	0.781

Note: Model 1: unadjusted model; Model 2: adjusted for age, gender, marriage, education, residence, co-residence, and income source; Model 3: adjusted for alcohol, smoking, exercise, chronic disease, ADL, denture use, social activities, and financial support from children based on Model 2.

**Table 3:** Multivariate logistic regression analysis between tooth loss and anxiety symptoms.

Characteristic	Reference	Model 4	*p*	Model 5	*p*	Model 6	*p*
		OR [95% CI]		OR [95% CI]		OR [95% CI]	
Tooth loose							
9–19	0–8	1.248 [1.031–1.512]	0.023	1.238 [1.017–1.507]	0.034	1.267 [1.037–1.548]	0.021
20–27	0–8	1.183 [0.990–1.413]	0.064	1.141 [0.941–1.383]	0.180	1.187 [0.973–1.449]	0.082
≥28	0–8	0.930 [0.777–1.113]	0.428	0.901 [0.739–1.098]	0.302	0.994 [0.799–1.237]	0.943
Age (years)							
≥80	65–79			0.718 [0.607–0.849]	<0.001	0.654 [0.550–0.777]	<0.001
Gender							
Female	Male			1.470 [1.261–1.713]	<0.001	1.386 [1.176–1.633]	<0.001
Marriage							
Others	Married			1.011 [0.852–1.200]	0.901	0.960 [0.806–1.143]	0.646
Education							
Yes	No			0.722 [0.615–0.847]	<0.001	0.751 [0.639–0.883]	<0.001
Residence							
Rural	City			1.357 [1.098–1.677]	0.005	1.418 [1.143–1.758]	0.001
Co-residence							
Alone	With family			1.282 [1.068–1.539]	0.008	1.382 [1.147–1.666]	<0.001
Nursing home	With family			1.634 [1.159–2.303]	0.005	1.510 [1.069–2.134]	0.019
Income source							
Family	Pensions			1.114 [0.901–1.377]	0.319	1.089 [0.879–1.350]	0.436
Others	Pensions			1.211 [0.969–1.512]	0.092	1.203 [0.961–1.507]	0.108
Alcohol							
Yes	No					0.805 [0.646–1.004]	0.055
Smoking							
Yes	No					0.940 [0.758–1.167]	0.577
Exercise							
Yes	No					0.845 [0.725–0.984]	0.030
Chronic disease							
Yes	None					1.648 [1.383–1.965]	<0.001
ADL							
Difficulty	No difficulty					0.751 [0.628–0.898]	0.002
Denture use							
Yes	No					0.759 [0.651–0.884]	<0.001
Social activities							
Sometimes	Never					0.933 [0.741–1.175]	0.556
Everyday						1.606 [1.102–2.340]	0.014
Financial support from children							
Yes	No					0.951 [0.695–1.303]	0.756

Note: Model 4: unadjusted model; Model 5: adjusted for age, gender, marriage, education, residence, co-residence, and income source; Model 6: adjusted for alcohol, smoking, exercise, chronic disease, ADL, denture use, social activities, and financial support from children based on Model 5.

**Table 4:** Mediation analysis of dietary diversity between tooth loss and depression/anxiety symptoms.

	Variable		Fitting Index			Significance of the Regression Equation			
Model	Independent Variable	Dependent Variable	R	R^2^	F	*β*	t	LLCI	ULCI
Model 1	Tooth loss	Depression symptoms	0.267	0.071	16.256	0.108	2.389 *	0.0194	0.1967
Model 2	Tooth loss	Dietary diversity	0.263	0.069	45.117	−0.057	−3.132 *	−0.093	−0.033
Model 3	Dietary diversity	Depression symptoms	0.278	0.077	16.160	−0.192	−7.138 ***	−0.245	−0.139
	Tooth loss					0.097	2.152 *	0.009	0.186
Model 4	Tooth loss	Anxiety symptoms	0.177	0.031	19.481	−0.006	−0.199	−0.066	0.054
Model 5	Tooth loss	Dietary diversity	0.263	0.069	45.117	−0.057	−3.132 **	−0.093	−0.021
Model 6	Dietary diversity	Anxiety symptoms	0.183	0.034	19.568	−0.082	−4.490 ***	−0.118	−0.046
	Tooth loss					−0.011	−0.352	−0.071	0.049

Note: controlled age, gender, marriage, education, residence, co-residence, income source, alcohol, smoking, exercise, chronic diseases, ADL, and denture use. * *p* ≤ 0.05, ** *p* ≤ 0.01, *** *p* ≤ 0.001.

## Data Availability

The data that support the findings of this study are openly available in the Chinese Longitudinal Healthy Longevity Survey (CLHLS) at https://doi.org/10.18170/DVN/WBO7LK (accessed on January 2024). Further inquiries can be directed to the authors.

## Supplementary Materials

The following supporting information can be downloaded at: https://www.mdpi.com/article/10.3390/nu18060893/s1, Table S1: Multivariate logistic regression analysis of tooth loss and depression symptoms by subgroups; Table S2: Multivariate logistic regression analysis of tooth loss and anxiety symptoms by subgroups.
