# Supplementary material for: The Effect of Tooth Loss on Depression and Anxiety Among Older Adults in China: The Mediating Role of Dietary Diversity
Source: Nutrients. 2026 Mar 12;18(6):893. doi: 10.3390/nu18060893 (PMC13028850; doi:10.3390/nu18060893)
Supplement: Supplementary file 1 [file nutrients-18-00893-s001.zip › nutrients-4164114-supplementary.pdf]

Supplementary Materials

The Effect of Tooth Loss on Depression and Anxiety Among Older Adults in China: The Mediating Role of Dietary Diversity

Yin Wang [1,2,3,4] and Xiaojie Sun [1,3,4,*]

[1] Department of Social Medicine and Health Management, School of Public Health, Cheeloo College of Medicine, Shandong University, Jinan 250012, China; wangyin21@mail.sdu.edu.cn
[2] School of Humanities and Management, Ningxia Medical University, Yinchuan 750001, China
[3] NHC Key Laboratory of Health Economics and Policy Research, Shandong University, Jinan 250012, China
[4] Center for Health Management and Policy Research, Shandong University (Shandong Provincial Key New Think Tank), Jinan 250012, China
* Correspondence: xiaojiesun@sdu.edu.cn; Tel.: +86-151-5316-9185

Table S1. Multivariate logistic regression analysis of tooth loss and depression symptoms by subgroups.

Model	Tooth loss 0–8	Tooth loss 9–19		Tooth loss 20–27		Tooth loss ≥28		*p* for interaction
		OR [95% CI]	*p*	OR [95% CI]	*p*	OR [95% CI]	*p*	
Age								
65–79	1(ref)	1.070 [0.815–1.405]	0.626	1.143 [0.848–1.541]	0.379	1.196 [0.847–1.688]	0.309	0.807
≥80	1(ref)	1.576 [1.158–2.146]	0.004	1.614 [1.217–2.140]	<0.001	1.388 [1.037–1.856]	0.027	
Gender								
Male	1(ref)	1.470 [1.090–1.983]	0.012	1.419 [1.051–1.916]	0.022	1.490 [1.080–2.056]	0.015	0.034
Female	1(ref)	1.117 [0.861–1.449]	0.407	1.260 [0.985–1.613]	0.066	1.000 [0.766–1.307]	0.997	
Education								
No	1(ref)	1.106 [0.813–1.505]	0.520	1.195 [0.907–1.573]	0.205	0.943 [0.703–1.263]	0.943	0.011
Yes	1(ref)	1.398 [1.082–1.807]	0.010	1.483 [1.133–1.939]	0.004	1.553 [1.160–2.078]	0.030	
Residence								
City	1(ref)	1.056 [0.706–1.578]	0.791	1.186 [0.787–1.787]	0.416	1.135 [0.901–2.163]	0.135	0.102
Rural	1(ref)	1.360 [1.083–1.707]	0.008	1.401 [1.127–1.742]	0.002	1.131 [0.893–1.432]	0.307	
Co-residence								
With family	1(ref)	1.432 [1.141–1.797]	0.002	1.410 [1.129–1.762]	0.002	1.248 [0.980–1.589]	0.072	0.935
Alone	1(ref)	0.979 [0.635–1.508]	0.922	1.068 [0.710–1.605]	0.753	0.968 [0.627–1.495]	0.883	
Nursing home	1(ref)	0.639 [0.229–1.786]	0.393	1.604 [0.619–4.153]	0.331	1.269 [0.458–3.514]	0.646	
Chronic disease								
No	1(ref)	1.356 [0.806–2.283]	0.251	1.736 [1.063–2.836]	0.028	1.662 [0.992–2.783]	0.054	0.559
Yes	1(ref)	1.245 [1.007–1.540]	0.043	1.283 [1.043–1.577]	0.018	1.099 [0.878–1.376]	0.411	
Denture use								
No	1(ref)	1.382 [1.102–1.733]	0.005	1.394 [1.109–1.753]	0.004	1.293 [0.994–1.681]	0.055	0.711
Yes	1(ref)	0.996 [0.663–1.497]	0.985	1.206 [0.839–1.735]	0.311	0.978 [0.691-1.385]	0.900	

Note: adjusted for age, gender, marriage, education, residence, co-residence, income source, alcohol, smoking, exercise, chronic diseases, ADL, and denture use.

Table S2. Multivariate logistic regression analysis of tooth loss and anxiety symptoms by subgroups.

Model	Tooth loss 0–8	Tooth loss 9–19		Tooth loss 20–27		Tooth loss≥28		*p* for interaction
		OR [95% CI]	*p*	OR [95% CI]	*p*	OR [95% CI]	*p*	
Age								
65–79	1(ref)	1.204 [0.928–1.562]	0.163	1.022 [0.758–1.378]	0.888	1.107 [0.791–1.548]	0.554	0.164
≥80	1(ref)	1.235 [0.893–1.708]	0.203	1.211 [0.901–1.627]	0.204	0.973 [0.714–1.326]	0.863	
Gender								
Male	1(ref)	1.498 [1.097–2.045]	0.011	1.230 [0.887–1.705]	0.215	1.176 [0.821–1.684]	0.376	0.261
Female	1(ref)	1.117 [0.861–1.450]	0.403	1.134 [0.882–1.459]	0.326	0.891 [0.677–1.173]	0.410	
Education								
No	1(ref)	1.137 [0.833–1.553]	0.418	1.111 [0.837–1.476]	0.465	0.978 [0.722–1.323]	0.884	0.273
Yes	1(ref)	1.339 [1.031–1.740]	0.029	1.228 [0.924–1.632]	0.158	0.995 [0.721–1.372]	0.973	
Residence								
City	1(ref)	1.250 [0.810–1.929]	0.313	1.203 [0.760–1.905]	0.430	1.147 [0.689–1.910]	0.597	0.619
Rural	1(ref)	1.274 [1.017–1.597]	0.035	1.194 [0.957–1.490]	0.115	0.962 [0.755–1.226]	0.754	
Co-residence								
With family	1(ref)	1.310 [1.040–1.649]	0.022	1.225 [0.974–1.540]	0.083	1.100 [0.856–1.412]	0.456	0.214
Alone	1(ref)	1.184 [0.763–1.837]	0.451	0.971 [0.629–1.499]	0.895	0.626 [0.384–1.021]	0.060	
Nursing home	1(ref)	0.744 [0.246–2.253]	0.601	1.320 [0.449–3.878]	0.614	1.353 [0.422–4.336]	0.611	
Chronic disease								
No	1(ref)	1.144 [0.690–1.896]	0.603	1.164 [0.712–1.901]	0.545	1.132 [0.674–1.900]	0.640	0.814
Yes	1(ref)	1.296 [1.042–1.611]	0.020	1.203 [0.968–1.494]	0.095	0.964 [0.758–1.227]	0.768	
Denture use								
No	1(ref)	1.308 [1.306–1.653]	0.024	1.200 [0.943–1.528]	0.139	1.080 [0.814–1.433]	0.594	0.135
Yes	1(ref)	1.045 [0.706–1.548]	0.826	1.010 [0.702–1.454]	0.957	0.817 [0.576–1.159]	0.258	

Note: adjusted for age, gender, marital status, education, residence area, co-residence, income source, alcohol consumption, smoking, exercise, chronic diseases, ADL, and denture use.
